# Screening High School Students for Eating Disorders: Results of a National Initiative

**Published:** 2008-09-15

**Authors:** S. Bryn Austin, Najat J. Ziyadeh, Sara Forman, Lisa A. Prokop, Anne Keliher, Douglas Jacobs

**Affiliations:** Division of Adolescent Medicine, Children’s Hospital, Boston, MA. Dr Austin is also affiliated with the Department of Society, Human Development, and Health, Harvard School of Public Health, Boston, Massachusetts.; Division of Adolescent Medicine, Children's Hospital, Boston, Massachusetts; Division of Adolescent Medicine, Children's Hospital, Boston, Massachusetts; Division of Adolescent Medicine, Children's Hospital, Boston, Massachusetts; National Eating Disorders Screening Program, Screening for Mental Health, Wellesley, Massachusetts; National Eating Disorders Screening Program, Screening for Mental Health, Wellesley, Massachusetts

## Abstract

**Introduction:**

Early identification and treatment of disordered eating and weight control behaviors may prevent progression and reduce the risk of chronic health consequences.

**Methods:**

The National Eating Disorders Screening Program coordinated the first-ever nationwide eating disorders screening initiative for high schools in the United States in 2000. Students completed a self-report screening questionnaire that included the Eating Attitudes Test (EAT-26) and items on vomiting or exercising to control weight, binge eating, and history of treatment for eating disorders. Multivariate regression analyses examined sex and racial/ethnic differences.

**Results:**

Almost 15% of girls and 4% of boys scored at or above the threshold of 20 on the EAT-26, which indicated a possible eating disorder. Among girls, we observed few significant differences between ethnic groups in eating disorder symptoms, whereas among boys, more African American, American Indian, Asian/Pacific Islander, and Latino boys reported symptoms than did white boys. Overall, 25% of girls and 11% of boys reported disordered eating and weight control symptoms severe enough to warrant clinical evaluation. Of these symptomatic students, few reported that they had ever received treatment.

**Conclusion:**

Population screening for eating disorders in high schools may identify at-risk students who would benefit from early intervention, which could prevent acute and long-term complications of disordered eating and weight control behaviors.

## Introduction

The acute and chronic medical and psychiatric consequences of eating disorders are well documented. Anorexia and bulimia nervosa are associated with comorbid medical conditions such as osteoporosis and complications of the gastrointestinal, cardiovascular, and endocrine systems (1-3). Binge eating disorder has been linked with psychiatric comorbidity and severe obesity (4). Compared with the general population, people with anorexia or bulimia nervosa are at increased risk of suicide (5,6). More prevalent forms of disordered weight control behaviors, such as vomiting and abuse of laxatives, are also associated with a range of negative health outcomes, such as esophagitis, gastric rupture, and impairment of digestive functioning (7-9). Additionally, disordered eating behaviors may be causally related to overweight and obesity (10-12).

In the United States, the lifetime prevalence of anorexia nervosa, bulimia nervosa, and binge eating disorder are estimated to be 0.9%, 1.5%, and 3.5%, respectively, among women and 0.3%, 0.5%, and 2.0%, respectively, among men (4). Men may make up 10%-25% of the population with anorexia nervosa or bulimia nervosa (4,13) and nearly half of cases of binge eating (4). Median age of onset for the 3 disorders is estimated to be 18-21 years (4).

Disordered weight control behaviors and symptoms that do not necessarily meet psychiatric criteria for an eating disorder diagnosis (7) are estimated to be as much as 20 times more common in community samples (14) than are those behaviors and symptoms that meet diagnostic criteria. In 2005 the Youth Risk Behavior Surveillance System (YRBSS) found that 6.2% of girls and 2.8% of boys reported vomiting or taking laxatives in the past month to lose or maintain weight (15). The Minnesota Student Survey of more than 81,000 high school students found that, among girls, in the past year, 8.8% vomited to control their weight, 1.9% used laxatives for weight control, and 25.6% reported binge eating; comparable estimates from this study for the 3 behaviors in boys were 1.6%, 1.7%, and 12.5%, respectively (16). The proportion of high school youth who report these behaviors that have been treated for their eating disorder symptoms is unknown.

Some studies have found a higher prevalence of disordered eating behaviors and attitudes among white girls than among girls of color, particularly African American girls, although others have reported varying results (16-23). The 2005 YRBSS documented a similar proportion of white and Latina high school girls who reported vomiting or using laxatives in the past month to control weight (6.7% and 6.8%, respectively); these behaviors were least commonly reported by African American girls (4%) (15). In the Minnesota Student Survey, compared with white girls, Latina and Asian girls, but not African American or American Indian girls, reported higher rates of disordered eating behaviors (16). In another school-based study, however, vomiting and laxative use to control weight were more common in African American than in white girls (24).

In research with boys, findings have been more consistent in documenting equal or higher risk in boys of color relative to white boys. In the 2005 YRBSS, Latino boys reported the highest rate (3.9%) of vomiting and laxative abuse in the past month, while a similar proportion of white (2.3%) and African American (2.8%) boys reported these disordered weight control behaviors in the past month (15). In the Minnesota Student Survey, compared with white boys, Latino, Asian, and American Indian boys, but not African American boys, reported higher rates of disordered eating and weight control behaviors (16). The Commonwealth Fund survey of more than 6,700 US youth in grades 5 through 12 found that African American and Latino boys reported higher rates of ever having binged and purged than did white boys (21).

Early identification and intervention for a range of mental health problems may reduce risk of progression of the illness, relapse, and comorbid conditions (25). A shorter period between symptom onset and start of treatment may improve prognosis for recovery from anorexia (26) and bulimia nervosa (27). Early detection through school-based screening can shorten the period between symptom onset and accessing care and help adolescents begin treatment at younger ages. Support staff in schools may be ideally situated to help identify at-risk youth of both sexes and all races/ethnicities, make referrals for clinical evaluation and treatment, and offer in-school support (28).

Working with staff in schools across the country in the winter of 2000, the National Eating Disorders Screening Program (NEDSP) coordinated, to our knowledge, the first-ever nationwide eating disorders screening initiative for high schools in the United States. The program was designed to promote early detection and treatment-seeking in adolescents with untreated eating disorder symptoms. NEDSP's parent organization, the national nonprofit organization Screening for Mental Health (http://www.mentalhealthscreening.org), has coordinated a number of national, broad-scale screening initiatives in schools, workplaces, communities, and the military that address depression, bipolar disorder, anxiety disorders, and alcohol abuse; it has also screened for eating disorders on college campuses (29). For the present analysis, our aims were to evaluate the screening program's ability to reach symptomatic youth who had not yet accessed treatment and to examine sex and racial/ethnic differences in symptoms and treatment history.

## Methods

### The NEDSP Program

NEDSP staff sent out registration information about the program by direct mail and e-mail to individual membership lists of national professional organizations for school psychologists, nurses, and counselors to invite high schools across the country to enroll in the program. Representatives from high schools then contacted NEDSP to enroll. All participating high schools were provided with a questionnaire to screen for student eating disorders; educational materials for use in classrooms or assemblies; and technical assistance to help staff implement the screening, handle student requests to discuss eating disorders, and make appropriate referrals for evaluation and treatment. NEDSP educational materials included a video and discussion guide, participatory classroom curriculum, and activity guide. All materials were designed to help motivate students to seek help with eating disorder symptoms. Care was taken to design materials that did not glamorize eating disorders or provide unnecessary details about disordered weight control methods. Educational content addressed healthy diet and activity, signs and symptoms to watch out for in friends and family, availability and efficacy of treatment, and the need to seek help for symptoms. In addition, materials offered students guidance on how to talk with a friend or family member who may have an eating disorder (30).

### Screening questionnaire

High schools administered the anonymous, self-report eating disorders screening questionnaire to students in classrooms and assemblies. The survey included the Eating Attitudes Test (EAT-26), a validated eating disorders screening instrument (31). Possible scores on the EAT-26 range from 0 to 78. A score of 20 or above indicates that a person may have an eating disorder and should be evaluated further by a mental health professional. The student screening questionnaire also included items that assessed how often in the past 3 months students had vomited to control their weight, engaged in eating binges, or exercised to lose or control their weight. Each of these behavioral questions was followed by 7 response options: never, less than once per month, 1-3 times per month, once per week, 2-6 times per week, once per day, and more than once per day. The item on vomiting was adapted from the YRBSS (32). The questionnaire included an item on past treatment for eating disorders and items on sex, age, race/ethnicity, height, and weight.

### Participants and sampling procedure

A total of 270 public, private, and parochial high schools signed up to participate in the screening program, and 152 schools from 34 states completed the screening and educational components of NEDSP. Ninety-eight schools returned more than 35,000 student screening forms for analysis. Because of cost constraints on data entry, a subset of student screening forms were randomly selected for analysis by using a 2-stage, clustered-sampling method. First, 33 schools were randomly sampled from the 98 that returned screening forms, then a random sample of forms was selected from these schools; the number of forms selected from a school was proportional to the number received from that school. Because of a change in protocol at the data entry site, 8 of the 33 schools had all of their surveys entered rather than a proportional random sample; therefore, weighting was used in analyses to adjust for the oversampling of student surveys from these 8 schools. This 2-stage selection procedure resulted in a sample of 5,740 screening forms.

### Variables and data analysis

Total EAT-26 scores were computed by adding individual item scores. For students who were missing 1 or 2 items on the EAT-26 (n = 272), total scores were scaled to values within the full possible range of the instrument. Students who were missing 3 or more items were excluded from analysis. A binary term for EAT-26 score was created on the basis of the recommended cutoff of a score of 20 as an indication of a possible eating disorder. Binary terms were created for each of the items on disordered behavior in the past 3 months: any report of vomiting to control weight, binge eating once a week or more, and exercising to lose or control weight more often than once per day.

Multivariate linear and logistic regression models were used to test sex and racial/ethnic group differences in mean EAT-26 scores and frequencies of reporting disordered eating and weight control behaviors and ever having been treated for an eating disorder. Multivariate models examining sex differences controlled for age and race/ethnicity, and models examining racial/ethnic group differences controlled for age and were stratified by sex.

In secondary analyses to explore whether symptom type and severity may explain sex differences in treatment history, we tested 4 multivariate models that estimated the odds of ever having been treated for an eating disorder, comparing girls with boys within each symptom-type subgroup of students, controlling for symptom severity. Thirty-nine students who did not respond to the eating disorders treatment history item were excluded. All models controlled for sex, age, race/ethnicity, and extreme thinness, which was included because it is a widely recognized sign and symptom of eating disorders (33) and is readily observable. Extreme thinness was classified according to the World Health Organization definition of grade 1 thinness as a body mass index (BMI) less than 18.5 kg/m^2^ in adults aged 18 years or older (34), then coded by using age- and sex-specific BMI values for ages younger than 18 years to correspond with the adult cutoff (35). Subsample restrictions and additional covariates included in each model were as follows: model 1, restricted to the subgroup of students (n = 518) with an EAT-26 score of 20 or higher, controlled for total EAT-26 score; model 2, restricted to the subgroup of students (n = 435) who reported vomiting to control their weight in the past 3 months, controlled for vomiting frequency; model 3, restricted to the subgroup of students (n = 366) who reported binge eating once per week or more in the past 3 months, controlled for binge frequency; model 4, restricted to the subgroup of students (n = 155) who reported exercising more than once per day to lose or control weight in the past 3 months, which is the highest severity level assessed for this item.

For all models, generalized estimating equation methods were used to account for the clustered study design by using SAS PROC GENMOD (SAS Institute Inc, Cary, North Carolina) (36). After 173 surveys were excluded because of missing data, the analytic sample included 5,567 students. Compared with students included in analyses, those excluded were less likely to describe themselves as white (*P* = .002) and more likely to have not reported a race/ethnicity (*P* < .001); we found no differences in age, sex, EAT-26 score, disordered eating or weight control behaviors, or past eating disorder treatment (*P* > .05). Analysis of NEDSP data was approved by the institutional review board at Children's Hospital Boston.

## Results

The sample included 58% (3,252) girls and 42% (2,315) boys; 3% (189) were African American, 2% (93) American Indian, 2% (134) Asian/Pacific Islander, 5% (303) Latino, 83% (4,629) white, and 4% (219) reported no ethnicity. The mean age was 15.9 (standard deviation 1.0) years. Girls were 3 to 5 times more likely than boys to score at or above the threshold on the EAT-26, to report vomiting to control their weight in the past 3 months, and to have ever been treated for an eating disorder (Table 1).

Among girls, few significant differences were found in eating disorder symptoms across racial/ethnic groups (Tables 2A and 2B). Compared with white girls, Latina girls were less likely and American Indian girls were more likely to score 20 or more on the EAT-26, and African American and American Indian girls were more likely to report exercising more than once per day to control their weight. In contrast, among boys, African American, American Indian, Asian/Pacific Islander, and Latino boys were consistently more symptomatic than were white boys across the range of disordered eating and weight control symptoms and behaviors.

Within symptom subgroups defined by EAT-26 score and binge eating, girls were roughly 3.5 times more likely to report that they had been treated for an eating disorder than were boys with comparable symptom severity (Table 3). Within the symptom subgroup defined by exercising once a day or more often to control weight, girls were almost 8 times more likely than boys to report having been treated for an eating disorder. In contrast, within the subgroup that reported vomiting, no sex difference was observed in the odds of having ever received treatment. In most models, extreme thinness was positively associated with the odds of having been treated for an eating disorder, but age and race/ethnicity were not, controlling for sex and symptom type and severity (data not shown).

## Discussion

NEDSP, to our knowledge the first national screening program for eating disorders held in high schools across the United States, found that almost 1 in 4 girls and 1 in 10 boys reported at least 1 disordered eating or weight control symptom serious enough to warrant further evaluation by a health professional. Applying these findings to the roughly 35,000 students who completed screening questionnaires, we estimate that close to 7,000 students with potential eating disorder symptoms were identified in participating schools. Furthermore, a large proportion of symptomatic students had never been treated for an eating disorder. Depending on the symptom type, the proportion of symptomatic students who had never received treatment was 83% to 95% of boys and 83% to 86% of girls. These results support 2 conclusions: 1) national screening for eating disorders in high schools reached a large number of students who were likely to have symptoms of disordered eating and weight control and 2) most symptomatic high school students were untreated. Coupled with evidence that early detection and intervention may improve treatment outcomes (26,37,38), these findings underscore the suitability of population screening (39) in high schools as a strategy to identify youth in need of clinical evaluation for eating disorders.

Findings relating to racial/ethnic group patterns are also informative. Among girls, we observed few differences in eating disorder symptoms across ethnic groups, a finding consistent with the national YRBSS (15) and Commonwealth Fund survey (21). Our findings differ from those reported in a meta-analysis of 35 studies, which included predominantly female participants aged 9 to 73 years; results of the meta-analysis document higher risk in white than in African American girls and women (22). That said, a second meta-analysis suggests that differences between African American and white girls and women may manifest for some symptom types (drive for thinness) but not for others (bulimia and binge eating) (23). Among boys, in almost every symptom category, whites reported lower rates than did any other ethnic group. These findings are consistent with results of the national YRBSS (15) and Commonwealth Fund survey (21) and the Minnesota Student Survey (16). Our study expands on the literature on adolescent boys by providing racial/ethnic group comparisons of eating disorder treatment history and of symptom severity with a validated eating disorder screening tool. NEDSP results indicate that school-based screening for eating disorders is appropriate and needed, both in schools that are racially/ethnically diverse and in those that are not diverse. In fact, these findings suggest that distributing screening resources differentially by sex or race/ethnicity would be inappropriate and possibly unethical; boys and girls of all races/ethnicities should be targeted.

Girls who participated in NEDSP were more than 4 times more likely than boys to have been treated for an eating disorder. Boys have a lower prevalence of disordered eating and weight control symptoms than do girls, and this difference may partly account for our finding. However, results of subgroup analyses of girls and boys with similar symptoms, controlling for symptom severity, age, race/ethnicity, and extreme thinness, suggest that differences in prevalence alone may not explain sex disparities in treatment history. As shown in Table 3, symptomatic boys were far less likely than girls to have accessed treatment. Our findings may overestimate the sex disparity in treatment history, possibly because of residual confounding as a result of incomplete control for sex differences in symptom severity. Alternatively, symptomatic boys may be overlooked by clinicians, school personnel, parents, and others because the prevalence of eating disorders in boys is commonly underestimated (40).

### Limitations

This study has several limitations. The sampling methods used to select screening forms for inclusion in the analysis allow our results to be generalizable to the larger pool of more than 35,000 screening forms received at NEDSP headquarters. However, students who participated in the screening program may not be representative of high school students as a whole in the United States. Schools that enrolled in the program and returned screening forms to NEDSP headquarters may have had greater resources, or their staff may have been more concerned about eating disorders than the staff of other schools. Despite these limits in generalizability, our estimate of the prevalence of eating disorder symptoms is comparable to that of a similarly designed screening study conducted with adolescent girls and young women aged 12 to 21 years who received routine care from a US military health care facility. In that sample, 21% of girls scored 20 or more on the EAT-26 or reported disordered eating or weight control behaviors (41); in NEDSP, 24.8% of girls met these criteria.

When controlling for symptom type and severity, we did not find race/ethnicity to be associated with the odds of ever having been treated for an eating disorder; however, because subgroup sizes were small, we may not have had sufficient power to detect disparities in treatment history. Prior research suggests that stereotypes may cause eating disorder symptoms to be underrecognized in African American and Latina girls (42). Furthermore, in a national eating disorder screening study carried out on college campuses in the United States, among those with eating disorder symptoms, nonwhite participants were less likely than white participants with comparable symptom severity to be asked about their symptoms by doctors and mental health professionals and less likely to receive a recommendation for further clinical evaluation (29).

The frequency and severity thresholds for each of the disordered eating and weight control symptoms were chosen to be clinically meaningful as a screening tool and were not designed to be diagnostic thresholds. Some students may have been incorrectly identified by the screening tool as having an eating disorder, and some may not have needed treatment. Despite the limitations of the thresholds selected for NEDSP, evidence suggests these cutoffs are meaningful. With a cutoff of 20 or higher, the sensitivity and specificity of EAT-26 are moderately high to high for detecting eating disorder cases that meet *Diagnostic and Statistical Manual of Mental Disorders, Fourth Edition* criteria, and when used as a dimensional measure, EAT-26 score is positively associated with severity of symptoms in women with subsyndromal eating disorders (43). In addition, a study in a nonpsychiatric sample of adults recommends a threshold of 11 on the EAT-26 to detect subsyndromal and EDNOS (eating disorder not otherwise specified) cases (44). In other work with the NEDSP sample, students who reported the disordered eating or weight control behaviors scored on average in the subthreshold (a score of 10 to 19) to threshold (a score of 20) range on the EAT-26; mean EAT-26 scores increased fairly linearly with increasing behavioral frequency for vomiting and binge eating (J. Haines, written communication). In addition, in female NEDSP participants, vomiting for weight control in the previous 3 months, even when infrequent, was associated with disruption of regular menstrual cycles (45).

Our analyses were based on self-report data, which are subject to bias resulting from cognitive and situational factors (46). Nevertheless, a validation study that used self-report to assess vomiting and laxative use for weight control in adolescent girls found high sensitivity (0.93) and specificity (0.86), although comparable estimates for a measure of binge eating were lower (sensitivity 0.53, specificity 0.78) (47).

### Conclusions

In June 2007, the US Senate directed the Centers for Disease Control and Prevention to intensify efforts to investigate the problem of eating disorders and their health implications for the US population (48). On the basis of results from the NEDSP screening initiative, we have identified a need for population screening and public health intervention in US high schools, since only a small fraction of students who self-identified as engaging in disordered eating and weight control behaviors had ever received treatment. For many of these adolescents, beginning treatment during high school or earlier would improve treatment effectiveness and mitigate acute and chronic complications of disordered eating and weight control behaviors, such as impaired growth and digestive functioning, osteoporosis, and obesity (4,26,37,38). NEDSP is a useful addition to the screening tools available to public health and school health practitioners to address this critical public health problem.

## Figures and Tables

**Table 1:** Eating Disorder Symptoms Among High School Students (N = 5,567) Participating in the National Eating Disorders Screening Program, 2000

Variable	Mean (SD) or %^a^		OR (95% CI)^b^

	Girls (n = 3,252)	Boys (n = 2,315)	
Weighted mean EAT-26 score (SD)^c^	9.5 (9.4)	4.4 (7.0)	NA
EAT-26 score ≥20, %	14.5	3.6	4.7 (3.3-6.6)
Vomiting to control weight in past 3 months, %	12.2	3.9	3.5 (2.1-5.9)
Binge eating ≥1 time/week in past 3 months, %	7.7	7.2	1.1 (0.8-1.6)
Exercising to control weight >1 time/day in past 3 months, %	3.4	2.8	1.3 (0.8-2.1)
Any symptom of disordered eating or weight control, %^d^	24.8	11.1	2.7 (2.1-3.4)
Ever been treated for an eating disorder, %	4.0	1.3	3.3 (1.8-6.0)

Abbreviations: SD, standard deviation; OR, odds ratio; CI, confidence interval; EAT-26, Eating Attitudes Test; NA, not applicable.

a All means and percentages are weighted.

b Multivariate models estimate odds of eating disorder symptoms associated with female sex, controlling for age and race/ethnicity.

c β coefficient (SE) = 5.2 (0.3). *P* < .001. Multivariate models estimate EAT-26 score associated with female sex, controlling for age and race/ethnicity.

d Defined as report of ≥1 of the following symptoms: EAT-26 score ≥20, vomiting to control weight in past 3 months, binge eating ≥1 time/week in past 3 months, exercise to control weight >1 time/day in past 3 months.

**Table 2A.** Eating Disorder Symptoms by Racial/Ethnic Group in High School Students (N = 5,567) Participating in the National Eating Disorders Screening Program, 2000

Race/Ethnicity	EAT-26 Score^a^		EAT-26 Score ≥20^a^		Vomiting^a^^,^ b	

	Mean	β (95% CI)	%	OR (95% CI)	%	OR (95% CI)
**Girls (n = 3,252)**						
African American (n = 115)	8.9	−0.7 (−3.9 to 2.5)	7.9	0.5 (0.2 to 1.2)	8.7	0.7 (0.3 to 1.5)
American Indian (n = 49)	12.5	3.0 (−1.9 to 7.8)	**25.7**	**2.0 (1.2 to 3.2)**	17.8	1.5 (0.7 to 3.3)
Asian/Pacific Islander (n = 77)	10.0	0.5 (−3.2 to 4.2)	13.8	0.9 (0.4 to 1.9)	16.1	1.4 (0.6 to 3.0)
Latina (n = 162)	**7.9**	**−1.7 (−2.9 to −0.4)**	**9.2**	**0.6 (0.4 to 0.9)**	9.3	0.7 (0.5 to 1.1)
White (n = 2,751)	9.5	1.0	14.7	1.0	12.4	1.0
No ethnicity reported (n = 98)	11.5	2.0 (−1.1 to 5.1)	19.2	1.4 (0.8 to 2.4)	10.4	0.8 (0.4 to 1.8)
**Boys (n = 2,315)**						
African American (n = 74)	5.7	1.7 (−1.0 to 4.4)	4.7	1.7 (0.7 to 4.1)	6.8	2.5 (0.8 to 7.2)
American Indian (n = 44)	8.5	4.4 (−1.6 to 10.5)	**11.8**	**4.6 (1.6 to 13.7)**	**11.8**	**4.5 (1.6 to 13.1)**
Asian/Pacific Islander (n = 57)	**7.8**	**3.8 (0.1 to 7.4)**	**8.3**	**3.1 (1.5 to 6.5)**	**10.9**	**4.0 (1.8 to 8.8)**
Latino (n = 141)	4.8	0.8 (−0.5 to 2.1)	5.2	1.9 (0.8 to 4.7)	5.4	1.9 (0.9 to 3.9)
White (n = 1,878)	4.0	1.0	2.8	1.0	2.9	1.0
No ethnicity reported (n = 121)	6.1	2.1 (−0.9 to 5.1)	**8.5**	**3.3 (1.2 to 8.7)**	**9.1**	**3.5 (1.3 to 9.3)**

Abbreviations: EAT-26, Eating Attitudes Test; CI, confidence interval; OR, odds ratio.

a All percentages are weighted. Multivariate models are stratified by sex and control for age and race/ethnicity. Values in **boldface** are significant at *P* < .05.

b Vomiting to control weight in the past 3 months.

**Table 2B.** Eating Disorder Symptoms by Racial/Ethnic Group in High School Students (N = 5,567) Participating in the National Eating Disorders Screening Program, 2000

Race/Ethnicity	Binge Eating^a^^,^ b		Frequent Exercise^a^^,^ c		Any Eating Disorder Symptoms^a^^,^ d		Ever Treated for an Eating Disorder^a^	

	%	OR (95% CI)	%	OR (95% CI)	%	OR (95% CI)	%	OR (95% CI)
**Girls (n = 3,252)**								
African American (n = 115)	9.6	1.3 (0.7-2.4)	**6.2**	**2.3 (1.1-4.8)**	21.9	0.8 (0.5-1.3)	2.9	0.7 (0.2-2.5)
American Indian (n = 49)	10.2	1.4 (0.7-2.7)	**15.2**	**6.2 (2.8-14.1)**	34.5	1.6 (1.0-2.6)	**15.6**	**4.4 (1.3-14.7)**
Asian/Pacific Islander (n = 77)	5.9	0.8 (0.3-1.9)	5.9	2.2 (0.7-6.3)	22.3	0.9 (0.4-1.8)	3.5	0.9 (0.2-3.2)
Latina (n = 162)	8.2	1.1 (0.5-2.6)	4.4	1.6 (0.7-3.5)	20.8	0.8 (0.5-1.2)	2.5	0.6 (0.2-2.1)
White (n = 2,751)	7.5	1.0	2.8	1.0	25.0	1.0	4.0	1.0
No ethnicity reported (n = 98)	13.3	1.9 (1.0-3.8)	**6.6**	**2.4 (1.1-5.5)**	23.8	0.9 (0.6-1.5)	3.8	1.0 (0.3-3.5)
**Boys (n = 2,315)**								
African American (n = 74)	**13.9**	**2.4 (1.3-4.5)**	**13.0**	**7.1 (4.0-12.6)**	**20.7**	**2.4 (1.5-3.8)**	0.9	0.8 (0.1-5.5)
American Indian (n = 44)	**19.3**	**3.5 (1.5-8.4)**	2.7	1.3 (0.1-12.0)	**22.0**	**2.6 (1.1-5.9)**	**5.3**	**5.0 (1.6-15.6)**
Asian/Pacific Islander (n = 57)	**16.9**	**2.9 (1.7-5.0)**	6.0	3.0 (0.9-9.8)	**19.9**	**2.2 (1.2-4.1)**	2.4	2.1 (0.4-11.3)
Latino (n = 141)	4.3	0.7 (0.3-1.2)	**4.8**	**2.4 (1.0-5.5)**	**15.3**	**1.7 (1.0-2.7)**	1.7	1.5 (0.5-4.9)
White (n = 1,878)	6.4	1.0	2.1	1.0	9.8	1.0	1.1	1.0
No ethnicity reported (n = 121)	9.6	1.6 (0.8-3.0)	**4.6**	**2.3 (1.1-4.9)**	12.5	1.3 (0.7-2.4)	1.5	1.4 (0.3-6.4)

Abbreviations: OR, odds ratio; CI confidence interval.

a All percentages are weighted. Multivariate models are stratified by sex and control for age and race/ethnicity. Values in **boldface** are significant at *P* < .05.

b Binge eating ≥1 time/week in the past 3 months.

c Exercising to control weight >1 time/day in the past 3 months.

d Defined as report of ≥1 of the following symptoms: EAT-26 score ≥20, vomiting to control weight in past 3 months, binge eating ≥1 time/week in past 3 months, exercise to control weight >1 time/day in past 3 months.

**Table 3:** Odds of Past Treatment for an Eating Disorder by Sex and Symptom Subgroup Among High School Students (N = 5,567) Participating in the National Eating Disorders Screening Program, 2000

Symptom Subgroup^a^	Girls		Boys(Referent)

	%^b^	OR (95% CI)	%^b^
Model 1: EAT-26 score ≥20 (n = 518)	**13.5**	**3.3 (1.1-10.1)**	**9.2**
Model 2: vomiting to control weight in past 3 months (n = 435)	16.5	1.9 (0.8-4.4)	16.9
Model 3: binge eating ≥1 time/week in past 3 months (n = 366)	**13.9**	**3.7 (1.7-7.6)**	**7.0**
Model 4: exercising to control weight >1 time/day in past 3 months (n = 155)	**15.2**	**7.9 (1.4-44.1)**	**4.6**

Abbreviations: OR, odds ratio; CI, confidence interval; EAT-26, Eating Attitudes Test.

a Multivariate models estimate odds of ever having been treated for an eating disorder associated with female sex, within each subset of symptomatic students. All models control for age, race/ethnicity, and extreme thinness (34). In addition, where possible, models control for symptom severity: model 1 controls for EAT-26 score, model 2 controls for vomiting frequency, and model 3 controls for binge frequency; for model 4, the variable definition is based on the highest exercise frequency available. Values in **boldface** are significant at *P* < .05.

b All percentages are weighted.
